# N-Terminal Metal-Binding Domain of Arabidopsis IBR5 Is Important for Its in Planta Functions

**DOI:** 10.3390/ijms26199315

**Published:** 2025-09-24

**Authors:** Jinouk Yeon, Jaebeom Lim, Sang-Kee Song, Hankuil Yi

**Affiliations:** 1 Department of Biological Sciences, Chungnam National University, Daejeon 34134, Republic of Korea; jwyeon93@gmail.com; 2 Department of Bio AI Convergence, Chungnam National University, Daejeon 34134, Republic of Korea; ljb1990a@naver.com; 3 Department of Biology, Chosun University, Gwangju 61452, Republic of Korea; sangkeesong@chosun.ac.kr; 4 Department of Convergent Bioscience and Informatics, Chungnam National University, Daejeon 34134, Republic of Korea

**Keywords:** IBR5, dual specificity phosphatase, rubredoxin-like domain, zinc

## Abstract

Indole-3-acetic acid (IAA), the predominant natural auxin, is a plant hormone that regulates growth and development in response to various internal and external signals. *Arabidopsis thaliana* (Arabidopsis) indole-3-butyric acid response 5 (*AtIBR5*, *AT2G04550*) encodes a dual-specificity phosphatase in Arabidopsis. The *atibr5* mutant exhibits reduced sensitivity to indole-3-butyric acid (IBA), a precursor of IAA, but is also less responsive to another plant hormone, abscisic acid (ABA). We report that AtIBR5 contains a rubredoxin-like domain in its N-terminal region, in addition to the previously identified dual-specificity phosphatase domain. The rubredoxin-like domain of AtIBR5, when expressed in *Escherichia coli*, binds zinc through four cysteine residues in the rubredoxin-like domain and exhibits a characteristic absorption spectrum at 430 nm. The rubredoxin-like domain, more specifically the set of four cysteine residues, is essential for most in planta functions of AtIBR5 related to auxin and ABA. These functions include hypocotyl elongation, leaf serration, and germination phenotypes. However, this domain is dispensable for the inhibition of root elongation by ABA. All orthologs of AtIBR5 in the green plant lineage investigated encode the N-terminal rubredoxin-like domain, which features the specific arrangement of four cysteine residues. Our result provides a clue as to how various plant phenotypes can be subtly modulated by AtIBR5.

## 1. Introduction

Auxin is a plant hormone involved in nearly every aspect of plant growth, development, and responses to various external stimuli [1,2,3]. It plays a crucial role in cell division, cell elongation, differentiation, tropism, and apical dominance, contributing to the development and regeneration of various tissues and organs. Besides plants, bacteria, fungi, and algae also produce auxin and use it as an internal or external signaling molecule [1,4,5]. In plants, auxin signaling primarily depends on the nuclear auxin pathway, which involves auxin co-receptor complexes and regulated transcription factors. However, non-transcriptional mechanisms also contribute to plant auxin signaling [3,6,7]. Phylogenomic studies on proteins involved in auxin metabolism, transport, and the nuclear auxin pathway suggest that a complete auxin response mechanism, including the nuclear auxin pathway, was established in basal land plants by utilizing pre-existing subdomains and transcriptional machinery [8,9].

The *Arabidopsis thaliana* (Arabidopsis) indole-3-butyric acid response 5 (*AtIBR5*, *AT2G04550*) encodes a dual-specificity phosphatase (DSP: EC 3.1.3.16 & 3.1.3.48; UniProt ID: Q84JU4). The atibr5 mutants were originally identified through screenings for mutants with attenuated responses to indole-3-butyric acid (IBA) and were also found to be less responsive to other auxins [10]. IBA serves as a precursor to indole-3-acetic acid (IAA), the predominant natural auxin, and is converted to IAA in peroxisomes [11]. The *atibr5* loss-of-function mutants exhibit several auxin-related phenotypes [10,12]. First, the primary roots of *atibr5* mutants are longer than those of wild-type (WT) plants, and the inhibition of root elongation by auxin is less pronounced in the mutants. Second, *atibr5* mutants display shorter hypocotyl lengths compared to WT plants. Third, the leaves of *atibr5* mutants exhibit more pronounced serrations, a trait influenced by the distribution and abundance of PIN-FORMED 1, an auxin efflux carrier [13]. Fourth, abnormal cotyledon vein patterns are more frequently observed in *atibr5* mutants. Additionally, *atibr5* mutants display reduced petal sizes due to a decrease in petal width [14]. The *atibr5* mutants also exhibit reduced responsiveness to another plant hormone, abscisic acid (ABA). Later studies revealed that AtIBR5 functions as a mitogen-activated protein kinase (MAPK) phosphatase, which inactivates MAPK12, and as a positive regulator of plant defense signaling through its interaction with the Toll/interleukin-1/Resistance domain [15]. However, the mechanism by which the activity of AtIBR5 is regulated has not been determined.

To successfully complete a normal life cycle, plants require mineral nutrients, which are components of metabolites or plant constituents, in addition to water, carbon dioxide, and oxygen [16,17]. Seventeen elements are generally considered essential nutrients and are traditionally classified as macronutrients or micronutrients, depending on whether they are required in large amounts (more than 10 mmol/kg of dry weight or 1000 mg/kg of dry weight). Mineral nutrients include various ionic forms of metals. Among macronutrients, potassium, calcium, and magnesium are essential, while iron, manganese, zinc, copper, and nickel are important micronutrients [18]. Some of these metal micronutrients play crucial catalytic and structural roles in plant proteins involved in metabolism, signal transduction, and gene regulation. For example, iron is redox-active and can exist as ferrous ions (Fe^2+^) or ferric ions (Fe^3+^), enabling various enzymes to facilitate oxygenation and electron transport [19]. Zinc ions (Zn^2+^), on the other hand, serve as redox-stable cofactors for enzymes such as oxidoreductases, hydrolases, and isomerases. Additionally, zinc functions as a structural component, notably in zinc finger domains, which are crucial for DNA binding and protein–protein interactions [20,21].

Here, we report that AtIBR5 possesses a rubredoxin-like domain at its N-terminal region, which is conserved in the green plant lineage and binds to zinc, and demonstrate that the domain is crucial for most, but not all, in planta functions of AtIBR5.

## 2. Results

### 2.1. AtIBR5 Contains a Putative Metal-Binding Rubredoxin-like Sequence in Its N-Terminal Region

**Figure 1:**
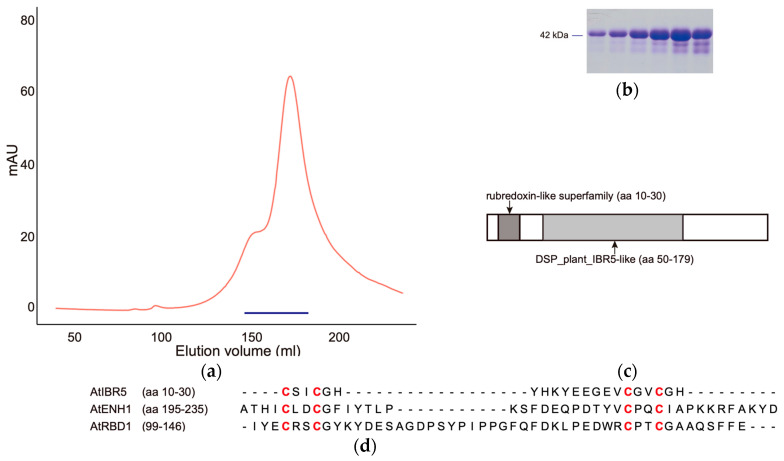
SUMO-AtIBR5 proteins exist as protein complexes. (**a**) Elution profile of SUMO-AtIBR5 analyzed via size exclusion chromatography. The red line represents the elution profile, showing relative absorption at 280 nm across the separation range of the column. AU: arbitrary units. The blue line indicates the fraction collection range. (**b**) SDS-PAGE analysis of the proteins collected in (**a**). Similar results were observed from more than three independent experiments. (**c**) Schematic representation of the two conserved domains identified in the AtIBR5 protein. (**d**) Sequence alignment of the rubredoxin-like domains in AtIBR5 (UniProt ID: Q84JU4), AtRBD1 (UniProt ID: Q9SLI4), and AtENH1 (UniProt ID: Q9FFJ2). The four conserved cysteine residues in each protein are highlighted in bold red. The “-” symbol indicates gaps introduced for sequence alignment.

To investigate whether AtIBR5 shares the ability to adopt multiple oligomeric states, similar to other dual-specificity phosphatases such as laforin and VH1, recombinant AtIBR5 was purified using affinity and size exclusion chromatography. For both laforin and VH1, dimerization has been suggested as essential for optimal catalytic function [22,23]. In the initial experiment, recombinant proteins were expressed in *Escherichia coli* as a fusion protein, SUMO-AtIBR5. This construct included an N-terminal six-histidine residue (6X His)-tagged small ubiquitin-related modifier protein (SUMO) translationally fused to the 257-amino acid (aa)-long AtIBR5, with a glycine residue linker in between. The SUMO-AtIBR5 protein, expected to have a monomeric molecular weight of approximately 42 kDa, was then purified using Ni-NTA affinity chromatography followed by size exclusion chromatography (SEC). Using a column capable of separating proteins over a wide range of sizes, SUMO-AtIBR5 eluted in two distinct peaks, in addition to a peak at an elution volume of around 100 mL that included various high-molecular-weight proteins exceeding the column’s capacity: the first at approximately 156 mL and the second at around 176 mL (Figure 1a,b). The purified proteins from both peaks were largely homogeneous and migrated similarly in SDS-PAGE (sodium dodecyl sulfate–polyacrylamide gel electrophoresis), consistent with the expected 42 kDa monomeric SUMO-AtIBR5. Based on size calibration experiments for the column, the proteins in the first peak were estimated to have a molecular weight of approximately 184 kilodaltons (kDa), consistent with a tetrameric form, while those in the second peak corresponded to approximately 87 kDa, suggesting a dimeric form. These results indicated that AtIBR5 DSP may also adopt multiple oligomeric states.

Unexpectedly, the proteins in both peaks displayed a brownish color, suggesting that the SUMO-AtIBR5 recombinant protein might have been bound to metal ions or cofactors (Figure S1) [24,25]. A conserved domain search for the AtIBR5 protein (E-value threshold = 0.02) revealed that it contains an N-terminal sequence (aa 10–30) similar to those found in the rubredoxin-like superfamily domain (Conserved Domains Database (CDD) accession number: cl00202) [26]. Additionally, it possessed a sequence similar to the DSP_plant_IBR5-like sequence (aa 50–179), which corresponded to the dual-specificity phosphatase domain of plant IBR5-like protein phosphatases (CDD accession number: cd18534) (Figure 1c). Rubredoxin is a non-heme-iron-containing protein in which four cysteine residues coordinate a single iron atom in the form of a ferrous ion [27]. To determine whether the N-terminal rubredoxin-like domain of AtIBR5 was responsible for the characteristic brown color, a truncated version of the protein, SUMO-AtIBR5-p, was generated. SUMO-AtIBR5-p consists of aa 48–257 of the AtIBR5, including only the DSP_plant_IBR5-like domain, and an N-terminal 6× HIS-SUMO tag with a glycine linker. The purified SUMO-AtIBR5-p protein did not exhibit the brown coloration observed in SUMO-AtIBR5, indicating that the rubredoxin-like domain (aa 10–30 of AtIBR5) was required for the color (Figure S1).

In Arabidopsis, two genes, *AtENH1* (*AT5G17170*) and *AtRBD1* (*AT1G54500*), have been reported to encode proteins containing rubredoxin-like domains [28,29]. Compared to the rubredoxin-like domains in these two Arabidopsis proteins, the rubredoxin-like domain in AtIBR5 was found to have fewer amino acids between the two ‘CXXC’ sequences (C: cysteine; X: any amino acid other than cysteine) (Figure 1d). In AtIBR5, the number of amino acids between the two ‘CXXC’ sequences was only 11, whereas in AtENH1 and AtRBD1, it was 18 and 29, respectively. It was unclear whether the intervening amino acids in AtIBR5 were sufficient to allow metal ion coordination by the four cysteine residues. To investigate this, the presence of protein structures containing two ‘CXXC’ sequences separated by 11 amino acids and capable of coordinating a metal ion was examined using the Protein Homology/analogY Recognition Engine V 2.0 (Phyre2) [30]. Supporting the hypothesis that an 11-amino acid separation was sufficient for metal binding, the crystal structures of ruberythrin (Protein Data Bank ID: 3PZA) and nigerythrin (Protein Data Bank ID: 1YUX) were identified [31,32]. In each structure, iron atoms were coordinated by four cysteine residues within two ‘CXXC’ motifs separated by 11 amino acids (Figure S2).

### 2.2. The Four Cysteine Residues in the Rubredoxin-like Domain Are Essential for the Characteristic Absorption Spectrum of AtIBR5

**Figure 2:**
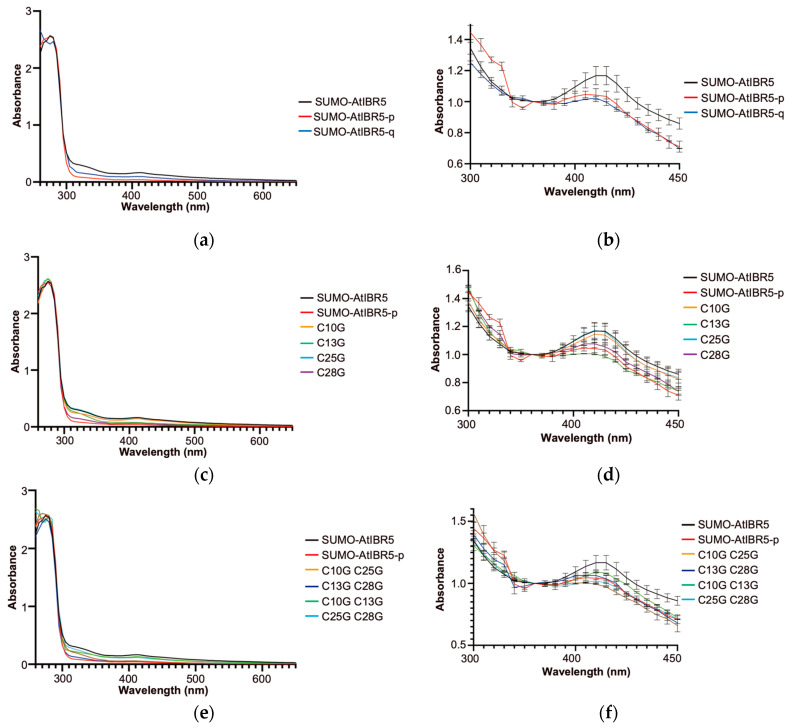
The absorption spectrum of various recombinant AtIBR5 proteins highlights the involvement of the four conserved cysteine residues. (**a**,**c**,**e**) Representative survey scan results across the ultraviolet and visible light spectrum (260–650 nm). (**b**,**d**,**f**) Survey scan results focusing on the absorption region around 420 nm. Each protein was scanned twice, and the average values with standard errors were plotted. SUMO-AtIBR5, SUMO-AtIBR5-p, and SUMO-AtIBR5-q are recombinant proteins carrying an N-terminal 6× His-SUMO tag and a glycine linker, translationally fused to full-length AtIBR5, an N-terminally truncated AtIBR5, and a quadruple AtIBR5 mutant, respectively. In SUMO-AtIBR5-q, a quadruple AtIBR5 mutant, all four conserved cysteine residues in the rubredoxin-like domain (C10, C13, C25, and C28) were substituted with glycine. The cysteine-to-glycine substitutions in SUMO-AtIBR5 mutant proteins are represented using the position numbers between “C” and “G.” For example, a single mutation at position 10 is denoted as C10G, while a double mutation at positions 10 and 25 is written as C10G C25G. The values were collected two or three times.

Rubredoxin proteins bind an iron atom using four cysteine residues and exhibit a characteristic absorption spectrum [33]. Similarly, the SUMO-AtIBR5 protein displayed an absorption spectrum around 430 nm wavelength, similar to those of rubredoxin domain-containing proteins, such as CrRBD1 in *Chlamydomonas reinhardtii* (Figure 2a) [34]. To investigate whether the four conserved cysteine residues in AtIBR5 play a crucial role in metal coordination and the resulting absorption spectrum, absorption patterns were compared across wavelengths from 260 nm to 650 nm between SUMO-AtIBR5 and SUMO-AtIBR5-p proteins purified. For further comparison, SUMO-AtIBR5-q, a quadruple-mutant in which all four conserved cysteine residues in SUMO-AtIBR5 were substituted with glycines, was also purified and analyzed. Mutant forms of AtIBR5 proteins, including SUMO-AtIBR5-p and SUMO-AtIBR5-q, displayed FPLC elution profiles with two major peaks corresponding to the size range of dimeric and tetrameric forms, similar to that of the SUMO-AtIBR5 wild-type protein. Proteins smaller than the expected size were also comparable to those observed for the wild-type protein in terms of both size and quantity (Figure 1 and Figure S3). While SUMO-AtIBR5 exhibited relatively broad peaks around 330 nm and 420 nm, the absorption at approximately 420 nm was significantly reduced in SUMO-AtIBR5-p, which lacks the N-terminal rubredoxin-like domain (Figure 2a,b). In terms of absorption patterns around 420 nm, SUMO-AtIBR5-q more closely resembled SUMO-AtIBR5-p than SUMO-AtIBR5. These results suggested that the four conserved cysteine residues are crucial for the characteristic absorption spectrum of SUMO-AtIBR5.

To further investigate the relative importance of each cysteine residue in the absorption spectrum, SUMO-AtIBR5 mutant proteins carrying single and selected double substitutions of the four conserved cysteines were analyzed. When each cysteine residue in SUMO-AtIBR5 was individually mutated to glycine, a more pronounced decrease in absorption around 420 nm was observed for C25G and C28G mutants (SUMO-AtIBR5 C25G and C28G) compared with C10G and C13G mutants (SUMO-AtIBR5 C10G and C13G) (Figure 2c,d). The absorption at 420 nm in C25G and C28G was reduced to a level comparable to that of SUMO-AtIBR5-p, which lacks the rubredoxin-like domain. When absorption at 420 nm was assessed in mutants with four of the six possible double cysteine substitutions, all tested double mutants (SUMO-AtIBR5 C10G C25G, C13G C28G, C10G C13G, and C25G C28G) exhibited absorption patterns more similar to SUMO-AtIBR5-p than to the SUMO-AtIBR5 (Figure 2e,f). Although the single C10G or C13G substitutions did not significantly reduce absorption at 420 nm (Figure 2d), the C10G C13G double mutant displayed a markedly reduced absorption peak at 430 nm compared with the wild type (Figure 2f).

To determine whether the putative rubredoxin-like domain can coordinate metal ions and, if so, whether it preferentially binds iron like rubredoxin, the metal content of recombinant SUMO-AtIBR5 was analyzed using inductively coupled plasma–mass spectrometry (ICP-MS). After expression in *Escherichia coli*, the proteins were first purified using Ni-NTA affinity chromatography via their N-terminal 6X His tag, followed by an additional SEC step for further purification. When the two-step-purified proteins were analyzed for metal content, SUMO-AtIBR5 contained similar amounts of zinc and nickel (Table 1, Figures S4 and S5). In contrast, the majority of metals detected in SUMO-AtIBR5-p and SUMO-AtIBR5-q were nickel. A similar nickel preference over zinc was observed in *Glycine max* cysteine desulfhydrylase 1 (GmDES1, UniProt ID: I1LBI3), which carries an N-terminal 6X His tag but lacks a metal-binding domain [25] (Table 1 and Figure S5). Given that all proteins analyzed by ICP-MS contained the same N-terminal 6X His tag and were purified using Ni-NTA affinity chromatography, the significantly higher zinc-to-nickel ratio in SUMO-AtIBR5 suggests that zinc is specifically bound by the rubredoxin-like domain via the four conserved cysteine residues. To rule out the possibility that the detected nickel and zinc were non-specifically bound during Ni-NTA affinity purification, additional ICP-MS analyses were performed after purifying SUMO-AtIBR5 and SUMO-AtIBR5-q without using Ni-NTA resin. To achieve this, MBP-AtIBR5 and MBP-AtIBR5-q—recombinant proteins carrying an N-terminal 6X His tag fused to maltose-binding protein (MBP)—were expressed and purified using amylose resin. After a second SEC purification step, ICP-MS analysis revealed that MBP-AtIBR5 contained approximately seven times more zinc than MBP-AtIBR5-q (Table 2 and Figure S4). No significant differences in the levels of the other five analyzed metals were observed between MBP-AtIBR5 and MBP-AtIBR5-q. Furthermore, the zinc content in AtIBR5 was found to be approximately ten times higher than nickel and twenty times higher than iron. These findings confirmed that AtIBR5 is a *bona fide* metal-binding protein and that its rubredoxin-like domain preferentially binds zinc over iron under the experimental conditions tested.

### 2.3. The Rubredoxin-like Domain in AtIBR5 Plays a Crucial Role in Various Auxin Responses

The *atibr5* mutant exhibits altered plant phenotypes associated with auxin-mediated responses, such as hypocotyl elongation, suppression of root elongation, leaf serration, and cotyledon vein development [10,12,13]. To determine whether the N-terminal rubredoxin-like domain is essential for the in planta function of AtIBR5 at the protein level, we generated transgenic *atibr5-3* plants that constitutively express AtIBR5 proteins either with or without a functional rubredoxin-like domain. Since the sufficient regulatory regions required for various AtIBR5-dependent plant phenotypes cannot be unequivocally determined, we chose to use the strong and constitutive cauliflower mosaic virus 35S (CaMV 35S) promoter. We hypothesized this approach would allow us to assess whether a specific form of AtIBR5 protein could complement a given phenotype at the protein level. The *atibr5-3* T-DNA insertional mutant (hereafter referred to as *atibr5*) is considered a loss-of-function mutant [35]. The *atibr5-3* mutant displays various auxin-related defects similar to those observed in the *atibr5-1* mutant, which carries a premature stop codon between the rubredoxin-like domain and the DSP_plant_IBR5-like domain [10,36]. The transgenic *atibr5*+*FL* line expressed a 3XFLAG-tagged full-length AtIBR5 protein under the control of the CaMV35S promoter. In contrast, *atibr5*+*p* and *atibr5*+*q* expressed only the DSP_plant_IBR5-like domain and a quadruple cysteine mutant form of AtIBR5 (C10G C13G C25G C28G), respectively. Both were transcriptionally regulated by the CaMV 35S promoter and tagged with 3XFLAG.

**Figure 3:**
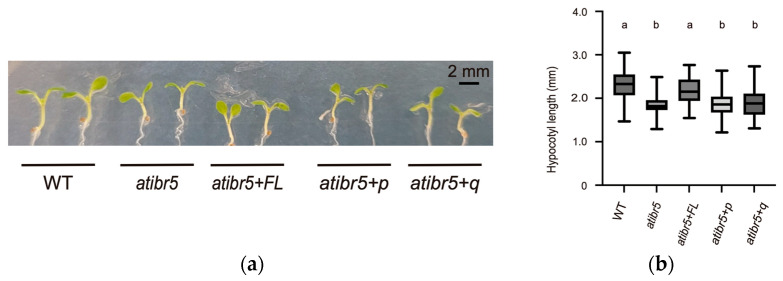
The full-length AtIBR5 transgene can complement the short hypocotyl phenotype of the *atibr5* mutant. (**a**) Representative images of hypocotyls in transgenic plants, wild type, and the *ibr5* mutant. WT (Col-0): wild-type Arabidopsis. *atibr5*: *atibr5* mutant (Salk_039359). *atibr5*+*FL*: atibr5 mutant expressing full-length AtIBR5. *atibr5*+*p*: *atibr5* mutant expressing only the DSP_plant_IBR5-like phosphatase domain of AtIBR5. *atibr5*+*q*: *atibr5* mutant expressing a mutant form of AtIBR5, in which the conserved four cysteine residues are substituted with glycines. (**b**) Hypocotyl lengths of transgenic plants, wild type, and the *ibr5* mutant. The hypocotyl lengths were measured twice. Statistical analysis was performed using a one-way ANOVA followed by Tukey’s post hoc test. Statistically significant differences are indicated by different letters (*p* < 0.05).

**Figure 4:**
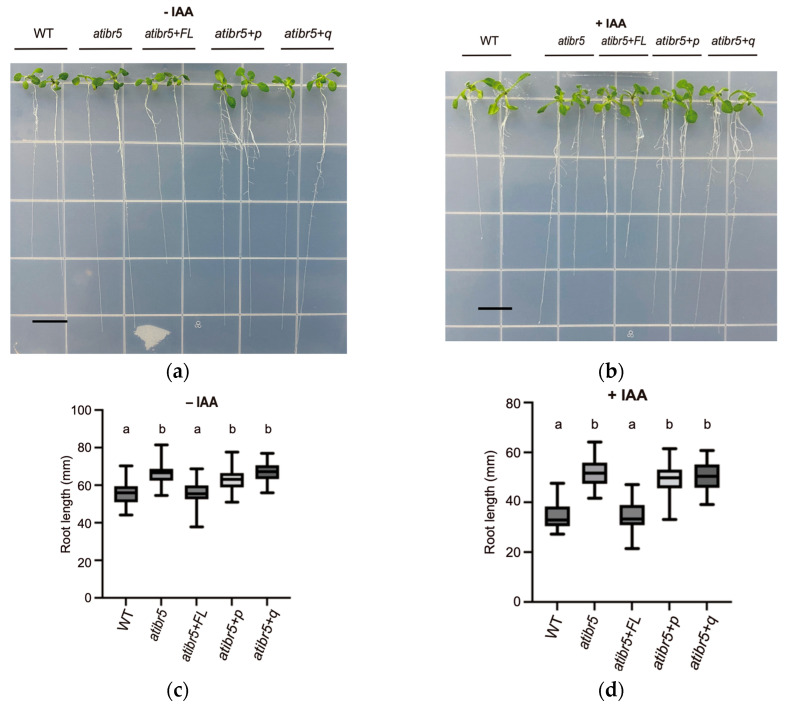
The longer primary root lengths observed in the *atibr5* mutant are not complemented by the expression of defective AtIBR5 proteins, regardless of IAA treatment. (**a**,**b**) Representative images of 10-day-old plants grown in the absence or presence of 100 nM IAA. WT (Col-0): wild-type Arabidopsis. *atibr5*: *atibr5* mutant (Salk_039359). *atibr5*+*FL*: atibr5 mutant expressing full-length AtIBR5. *atibr5*+*p*: *atibr5* mutant expressing only the DSP_plant_IBR5-like phosphatase domain of AtIBR5. *atibr5*+*q*: *atibr5* mutant expressing a mutant form of AtIBR5, in which the conserved four cysteine residues are substituted with glycines. Scale bar = 1 cm. (**c**,**d**) Root lengths of 10-day-old plants grown in the absence or presence of 100 nM IAA. Statistical analysis was performed using a one-way ANOVA, followed by Tukey’s post hoc test with a significance level of *p* = 0.01. Groups labeled with ‘a’ are significantly different from groups labeled with ‘b’. The same grouping pattern was observed in two independent experiments.

The functional rubredoxin-like domain is essential for restoring the auxin response. As previously reported, *atibr5* mutants exhibit shorter hypocotyls than WT plants [10] (Figure 3). Statistical analysis of hypocotyl length revealed that plants with different genotypes could be categorized into two distinct groups, indicating that a functional rubredoxin-like domain in AtIBR5 is necessary for complementing the short hypocotyl phenotype in *atibr5*: WT plants and *atibr5*+*FL* displayed significantly longer hypocotyls, while *atibr5*, *atibr5*+*p*, and *atibr5*+*q* exhibited shorter hypocotyls (Figure 3). Notably, in *atibr5*+*p* and *atibr5*+*q* plants, which failed to complement the short hypocotyl phenotype, FLAG-tagged AtIBR5 mutant proteins were expressed at levels comparable to those in *atibr5*+*FL* (Figure S6). The requirement of the rubredoxin-like domain in AtIBR5 was also evaluated for primary root growth and auxin-mediated inhibition of root elongation [10,12]. In the absence of IAA, the root lengths of *atibr5*, *atibr5*+*p*, and *atibr5*+*q* were similar to one another but significantly longer than those of WT plants. However, the root lengths of atibr5+FL were comparable to those of WT plants under normal conditions (Figure 4a). Upon treatment with 100 nM IAA, root elongation in *atibr5*, *atibr5*+*p*, and *atibr5*+*q* was inhibited to a similar extent, while the inhibitory effect in *atibr5*+*FL* was comparable to that observed in WT plants (Figure 4b).

**Figure 5:**
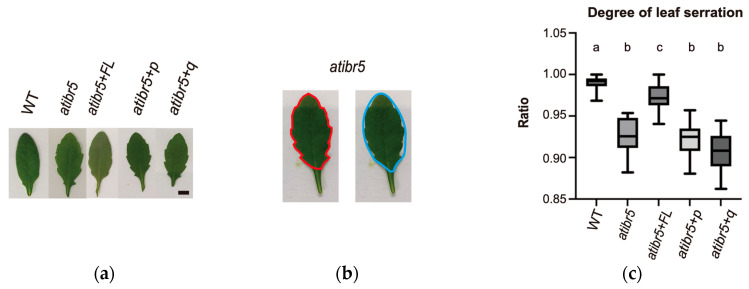
The leaf serration phenotype in *atibr5* cannot be complemented by AtIBR5 proteins with defective rubredoxin-like domains. (**a**) Representative leaf images of WT, *atibr5*, and *atibr5* transgenic plants expressing either intact or defective rubredoxin domains. WT (Col-0): wild-type Arabidopsis. *atibr5*: *atibr5* mutant (Salk_039359). *atibr5*+*FL*: atibr5 mutant expressing full-length AtIBR5. *atibr5*+*p*: *atibr5* mutant expressing only the DSP_plant_IBR5-like phosphatase domain of AtIBR5. *atibr5*+*q*: *atibr5* mutant expressing a mutant form of AtIBR5, in which the conserved four cysteine residues are substituted with glycines. Scale bar = 1 cm. (**b**) Outlines for measuring two areas, one real (shown in red) and one imagined (shown in blue), to compare the degrees of leaf serration. (**c**) A box plot showing the ratios of the two areas measured for each genotype. Leaf areas were measured three times. Statistical analysis was performed using one-way ANOVA followed by Tukey’s post hoc test. Statistical differences are indicated by different letters (*p* < 0.01).

*atibr5* displays more pronounced leaf serrations [10]. In *atibr5*+*FL*, a transgenic *atibr5* plant expressing the 3XFLAG-tagged full-length AtIBR5 under the control of the 35S promoter, the leaf serration phenotype was ameliorated compared to *atibr5* mutant (Figure 5a). However, leaf serration phenotype in *atibr5*+*p* or *atibr5*+*q* transgenic plants, which expresses AtIBR5 proteins only with the phosphatase domain without the N-terminal rubredoxin-like domain or the quadruple cysteine mutant form (C10G C13G C25G C28G), was comparable to that of *atibr5* mutant. The degree of leaf serration was quantitatively compared among WT, *atibr5*, *atibr5*+*FL*, *atibr5*+*p*, and *atibr5*+*q*, by measuring the ratio of the actual leaf area over the area inside the imagined elliptical boundary, which is formed by connecting all the serrated edges of a leaf (Figure 5b). We reasoned that more sharply serrated leaves have larger differences between two areas. Degrees of leaf serration in WT and *atibr5* were found to be statistically different from each other, confirming that this method can be used to compare degrees of leaf serration quantitatively (Figure 5c). This method revealed that degree of leaf serration in *atibr5*+*p* or *atibr5*+*q* was similar to that in atibr5 mutant. Although degree of leaf serration in *atibr5*+*FL* was found to be statistically different from that in *atibr5*, it was also different from that in WT. These results suggested that the rubredoxin-like domain is required for the complementation of leaf serration phenotype in *atibr5*.

**Figure 6:**
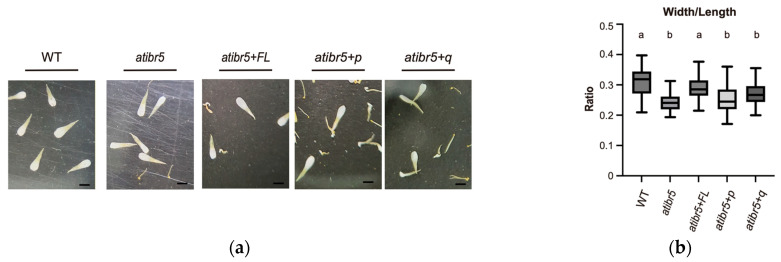
Functional rubredoxin-like domain in AtIBR5 is necessary for normal petal growth. (**a**) Representative images of petals used in the analysis, collected at the point of natural abscission along with other floral organs. WT: Col-0 wild-plants. *ibr5*: *ibr5-3* mutant. *ibr5*+*FL*: transgenic *ibr5* plant expressing full-length AtIBR5 WT. *ibr5*+*p*: transgenic *ibr5* plant expressing truncated AtIBR5. *ibr5*+*q*: transgenic *ibr5* plant expressing quadruple cysteine mutant form of AtIBR5. Scale bar = 1 mm. (**b**) Ratios of petal width to petal length in plants of different genotypes. Petal lengths and widths were measured three times. Statistical analysis was performed using one-way ANOVA followed by Tukey’s post hoc test. Significant differences are indicated by different letters (*p* < 0.01).

**Figure 7:**
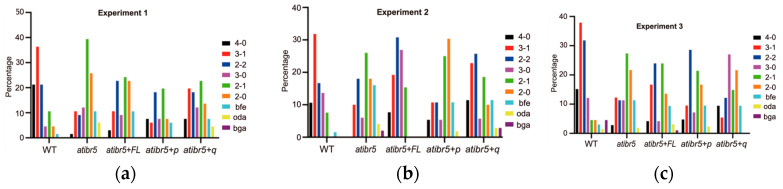
Distribution of cotyledon vein patterns in WT, atibr5 mutant, and transgenic lines. (**a**–**c**) Distribution of vein patterns according to Yanagisawa et al. (2021) [38] across three independent experiments. WT: Col-0 wild-plants. *ibr5*: *ibr5-3* mutant. *ibr5*+*FL*: transgenic *ibr5* plant expressing full-length AtIBR5 WT. *ibr5*+*p*: transgenic *ibr5* plant expressing truncated AtIBR5. *ibr5*+*q*: transgenic *ibr5* plant expressing quadruple cysteine mutant form of AtIBR5.

Defects in AtIBR5 also result in the reduction in organ sizes, especially the petal sizes, and AtIBR5 was proposed to regulate plant growth through auxin responses and TCP transcription factors [14]. Transgenic expression of full-length AtIBR5 restored the width/length ratio of *atibr5* petals to WT level, but no significant effects were observed when the DSP_plant_IBR5-like phosphatase domain was expressed without the rubredoxin-like domain or with the quadruply mutated rubredoxin-like domain (Figure 6). In addition, the importance of polar auxin transport in cotyledon vein formation has also been established [37]. When vein patterns were classified using the method described by Yanagisawa et al. [38], only the 4-0, 3-0, and 2-1 patterns—among six total patterns based on cotyledon complexity—showed statistically significant differences between WT and atibr5 (Figure 7 and Figure S6). However, even the introduction of the full-length AtIBR5 construct, let alone the other two mutant constructs, failed to consistently restore *atibr5* cotyledon defects. Our results showed that overexpression of AtIBR5 with both the functional rubredoxin-like domain and DSP_plant_IBR5-like phosphatase in AtIBR5 is required for all auxin-related phenotypes tested, except the cotyledon vein development.

### 2.4. The Rubredoxin-like Domain in AtIBR5 Is Required for ABA-Mediated Suppression of Germination but Dispensable for ABA-Mediated Root Elongation

**Figure 8:**
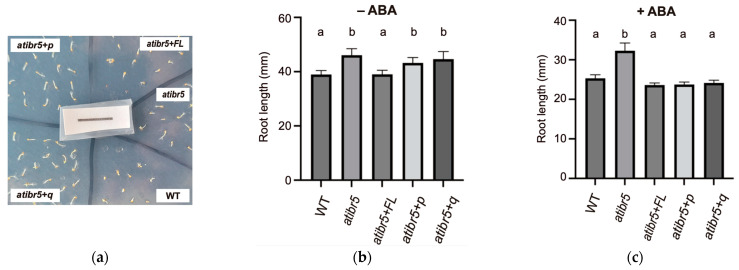
Phenotypes and root elongation of plants grown on solid media supplemented with ABA. (**a**) Plants grown for two days in the absence of ABA. WT: Col-0 wild-plants. *ibr5*: *ibr5-3* mutant. *ibr5*+*FL*: transgenic *ibr5* plant expressing full-length AtIBR5 WT. *ibr5*+*p*: transgenic *ibr5* plant expressing truncated AtIBR5. *ibr5*+*q*: transgenic *ibr5* plant expressing quadruple cysteine mutant form of AtIBR5. (**b**,**c**) Root length of 7-day-old plants: Plants were grown for four days on solid media supplemented with 10 μM ABA, following an initial growth period without ABA for three days. The values were obtained from three independent experiments. Statistical analysis was performed using a one-way ANOVA, followed by Tukey’s post hoc test. Statistically significant differences are indicated by different letters (*p* < 0.01).

In addition to auxin, the *atibr5* mutant exhibits reduced sensitivity to another plant hormone, ABA. As a result, *atibr5* mutants germinate slightly earlier than WT and display reduced sensitivity to ABA-mediated suppression of root elongation [10]. To assess seed germination, the emergence of radicles from imbibed seeds was examined in the absence of ABA. It was observed that *atibr5* germinated earlier than WT, whereas *atibr5*+*FL* germination was similar to that of WT plants (Figure 8a). In contrast, *atibr5*+*p* and *atibr5*+*q*, both carrying defective rubredoxin-like domains, germinated similarly to the *atibr5* mutant—earlier than WT and *atibr5*+*FL*.

Since ABA influences both seed germination and root elongation, we investigated its role in ABA-mediated suppression of root elongation by measuring root growth over a four-day period in the presence or absence of ABA, following an initial three-day growth phase in ABA-free media. In the absence of ABA, atibr5 mutants exhibited longer roots than WT. Only the expression of full-length AtIBR5 with an intact rubredoxin-like domain successfully restored the root length phenotype of *atibr5* (Figure 8b). However, in the presence of ABA, *atibr5*+*p* and *atibr5*+*q* mutants showed significantly reduced root growth, comparable to WT and *atibr5*+*FL* (Figure 8c). These results indicated that ABA-mediated suppression of root elongation could be restored by the DSP_plant_IBR5-like phosphatase domain alone. These findings suggested that the rubredoxin-like domain in AtIBR5 has distinct functional requirements in ABA-mediated germination and suppression of root elongation, playing a crucial role in the former but being dispensable for the latter.

## 3. Discussion

### 3.1. Orthologs of AtIBR5 Are Found Exclusively in the Green Plant Lineage, Including Chlamydomonas Reinhardtii and Physcomitrium Patens

Among the 22 DSPs in Arabidopsis, AtIBR5 is unique in possessing an N-terminal rubredoxin-like domain [39] (Figure 1c). Orthologs of AtIBR5, carrying plant IBR5-like protein phosphatases domain (cd18534), are conserved across various plants and algae. In the Protein Analysis Through Evolutionary Relationships (PANTHER) database (http://pantherdb.org, accessed on 5 February 2025), a publicly available knowledgebase providing insights into the evolution of protein-coding gene families, AtIBR5 orthologs were identified in all 39 fully sequenced plant genomes, including Amborella, sunflower, and spinach (Figure S7). Notably, 14 species were found to have more than one ortholog, likely due to whole-genome duplications, which are common in plants [40]. Multiple sequence alignment of representative IBR5 proteins—one from each of the 39 species—clearly demonstrated that these proteins possess a conserved DSP domain, along with a high degree of sequence similarity in their N-terminal regions, as previously reported (Figure S7) [10]. Across all orthologs, including those in Chlorophyta and Charophyta, the conserved N-terminal sequences are characterized by two CXXC motifs separated by 11 amino acids, suggesting that the rubredoxin-like domain plays important roles in the function of plant IBR5. However, no orthologs were found outside the green plant (Viridiplantae) lineage (Figure S8). The rubredoxin-like domain, featuring two CXXC motifs, fused to the DSP domain was exclusive to the green plant lineage, although DSP domains were also identified in red algae, brown algae, and even in an animal species, reflecting the early evolutionary presence of protein phosphatases in eukaryotes and MAP kinase signaling pathway [39,41]. In Arabidopsis, AtIBR5 has been shown to function in auxin and ABA responses, as well as in the mitogen-activated protein kinase (MPK) signaling pathway through the dephosphorylation of AtMPK12 [10,15]. Auxin and ABA have been reported to play physiological roles in red and brown algae, and the MPK signaling pathway is conserved across eukaryotes [15,42]. The presence of rubredoxins in photosynthetic algae and plants, as well as in diverse bacterial and archaeal groups, suggests that IBR5 evolved a novel function in the green plant lineage through the fusion of rubredoxin-like and DSP domains [28].

### 3.2. Zinc Binds to AtIBR5 Through Four Cysteine Residues in Its N-Terminal Rubredoxin-like Domain, When Expressed in E. coli

The four cysteine residues—C10, C13, C25, and C28—are responsible for the characteristic absorbance spectrum and most of the physiological functions of AtIBR5. Although AtIBR5 contains an additional partially overlapping CXXC sequence that includes C28 and C31, we do not consider C31 essential for AtIBR5 functionality. The SUMO-AtIBR5-q mutant with C31 exhibited an absorption spectrum similar to that of SUMO-AtIBR5-p mutant at around 430 nm (Figure 2b). Furthermore, transgenic overexpression of AtIBR5-q in the *atibr5* mutant failed to complement most mutant phenotypes associated with auxin and ABA responses (Figure 3, Figure 4, Figure 5, Figure 6 and Figure 8a). Sequence alignment of AtIBR5 orthologs also revealed that no other IBR5 protein possesses a cysteine residue at the corresponding position (Figure S8). However, it remains possible that C31 partially contributes to AtIBR5 function, although unequal contributions from C10, C13, C25, or C28 cannot be ruled out. While the absorption spectrum of the C28G mutant resembled that of SUMO-AtIBR5-p, the C25G mutant exhibited a spectrum similar to that of SUMO-AtIBR5, suggesting that C25 is dispensable (Figure 2d).

The preferential binding of AtIBR5 to zinc was demonstrated using recombinant proteins, which were affinity-purified with Ni-NTA or amylose resin following heterologous expression in *E. coli*. Although elevated nickel levels were detected in SUMO-AtIBR5, SUMO-AtIBR5-p, and SUMO-AtIBR5-q compared to the size exclusion chromatography buffer, this enrichment likely resulted from the presence of an N-terminal 6X His tag in the protein and the use of nickel during purification (Figure S5). Similarly, GmDES1, which also contains a 6X His tag but lacks the rubredoxin-like domain, exhibited preferential nickel binding (Table 1 and Figure S5) [25]. Notably, MBP-AtIBR5, which was purified using amylose resin, displayed significant zinc enrichment compared to MBP-AtIBR5-q (Table 2). Although natural and artificially designed rubredoxins are typically found in iron-bound forms, recombinant AtIBR5 proteins purified from *E. coli* in this study preferentially bound zinc over iron (Table 1 and Table 2) [27,43]. It is possible that the rubredoxin-like domain in AtIBR5 can bind various divalent metals, and the enrichment of zinc in AtIBR5 may be influenced by cellular zinc availability during overexpression under our experimental conditions. This hypothesis is supported by the following reports: both iron-containing and zinc-containing rubredoxins were isolated through the heterologous expression of *Clostridium pasteurianum* rubredoxin in *E. coli*. Additionally, five metals—copper, nickel, gallium, cadmium, and mercury—were shown to substitute for iron in *C. pasteurianum* rubredoxin [44,45]. However, we do not rule out the possibility that AtIBR5 has an intrinsic binding preference for zinc over iron. It has been reported that *E. coli* cells accumulate approximately equal amounts of zinc and iron when grown in Luria–Bertani broth [46]. Further investigation is needed to determine whether AtIBR5 proteins exhibit a preference for zinc binding in Arabidopsis and, if so, how this specificity is maintained.

### 3.3. Rubredoxin-like Domain in AtIBR5 Is Required for Complementation of Most atibr5 Mutant Phenotypes

By comparing the complementation phenotypes of *atibr5* transgenic plants expressing the AtIBR5 phosphatase domain with or without the functional N-terminal rubredoxin-like domain, we found that the requirement for the N-terminal domain depends on the specific phenotype investigated but the requirement is independent of the hormone involved. The full-length AtIBR5, including its functional N-terminal domain, was required for the complementation of various *atibr5* mutant phenotypes. These included the short hypocotyl phenotype (Figure 3), increased primary root length (Figure 4a), inhibition of primary root elongation by auxin (Figure 4b), enhanced leaf serration (Figure 5), a reduced petal width-to-length ratio (Figure 6), and accelerated germination (Figure 8a). However, the rubredoxin-like domain was found to be dispensable for the inhibition of primary root elongation by ABA (Figure 8c). The cotyledon vein pattern defects were not fully rescued, even by transgenic overexpression of the full-length AtIBR5 construct (Figure 7). Since the formation of cotyledon vein patterns requires spatially regulated developmental processes governed by polar auxin transport [37], the lack of complementation might be due to the use of the constitutive, non-cell-type-specific CaMV 35S promoter. Interestingly, while the N-terminal rubredoxin-like domain was dispensable for the inhibition of primary root elongation by ABA, it was required for the same response by auxin. This suggests that the inhibitory effects of ABA and auxin might be subtly influenced by the presence of this domain (Figure 4b and Figure 8c). For AtIBR5, both MPK phosphatase and holdase activities have been reported as molecular functions or enzymatic activities [15,36]. The differential requirement for the N-terminal domain in AtIBR5 function may depend on the specific enzymatic activity utilized and/or the interacting protein substrate.

## 4. Materials and Methods

### 4.1. Plant Materials and Arabidopsis Transformation

*Arabidopsis thaliana* (Arabidopsis) Columbia accession (Col-0) plants were grown under long-day conditions (16 h light/8 h dark) with a light intensity of 140 µmol/m^2^/s at 23 °C. A homozygous atibr5-3 mutant was selected from the progeny of Salk_039359 obtained from ABRC. Primer sequences used to identify the homozygous line are provided in Table S1. Transgenic plants were generated using the floral dip method with Agrobacterium tumefaciens GV3101 carrying IBR5 constructs cloned into the pCAMBIA1390 vector [47]. T1 transformants and their self-pollinated progeny were selected based on hygromycin resistance (Merck KGaA, Darmstadt, Germany). For each construct, at least three independent homozygous T3 transgenic lines were selected, and one representative line per construct was used for subsequent measurements.

### 4.2. Cloning and Mutagenesis

For protein purification, full-length and various mutant forms of AtIBR5 coding sequences were cloned using the Expresso T7 SUMO cloning and expression system (Lucigen, Middleton, WI, USA), as described by Hyun et al. [48]. While SUMO-AtIBR5 includes the full-length coding sequence of AtIBR5, SUMO-AtIBR5-p contains only the coding sequence starting from the dual-specificity phosphatase domain corresponding to amino acids 48–257 of AtIBR5. For efficient cleavage of the 6× His-SUMO tag after purification, a ‘GGU’ nucleotide sequence encoding a glycine linker was inserted between the sequences for 6× His-SUMO and the various forms of AtIBR5 proteins. Coding sequences for single-mutant proteins were generated by site-directed mutagenesis PCR, using the full-length AtIBR5 coding sequence cloned in the pETite N-His SUMO Kan vector as a template. Double- and quadruple-mutant coding sequences were constructed by sequentially introducing point mutations, using single- or double-mutant sequences as templates. Mutagenesis was performed using the QuickChange kit (Agilent Technologies, Santa Clara, CA, USA), and each intended sequence change introduced by mutagenesis PCR was confirmed using the dideoxy sequencing method. For in planta functional complementation of AtIBR5 mutant phenotypes, the full-length, phosphatase domain, and quadruple mutants of AtIBR5-coding sequences were cloned into the modified pCAMBIA1390 vector. This vector was modified to express cloned genes with a C-terminal 3× FLAG tag, under the control of the CaMV 35S promoter. Primer sequences used for PCR amplification and mutagenesis are provided in Table S1.

### 4.3. Expression and Affinity Purification of Recombinant Proteins

BL21 HiControl cells containing the desired expression plasmids were cultured in terrific broth, which consists of nutrients (12 g/L tryptone, 24 g/L yeast extract, and 0.4% glycerol) and a phosphate buffer (9.4 g/L potassium phosphate dibasic and 2.2 g/L potassium phosphate monobasic), supplemented with appropriate antibiotics, at 37 °C. When the optical density at 600 nm (OD_600_) reached 0.6–0.7, isopropyl β-D-1-thiogalactopyranoside was added to a final concentration of 0.5 μM. Following overnight incubation at 20 °C, cells were harvested by centrifugation at 4000× *g* for 10 min at 4 °C. For Ni-NTA affinity purification, harvested cells were resuspended in lysis buffer (50 mM Tris-HCl, pH 8.0, 500 mM NaCl, 20 mM imidazole, 10% glycerol, and 0.1% Tween-20) and disrupted using an XL 2020 sonicator (Misonix, Farmingdale, NY, USA) for eight cycles, each consisting of 2 min intervals with alternating 1 s on and 1 s off pulses. The soluble fraction was separated by centrifugation at 12,000× *g* for 40 min at 4 °C. The 6× His-tagged protein was captured by passing the soluble fraction through an Econo-Pac^®^ Chromatography Column (Bio-Rad, Hercules, CA, USA) packed with PureCube 100 Ni-NTA Agarose (Cube Biotech, Monheim, Germany). The resin was washed with wash buffer (50 mM Tris-HCl, pH 8.0, 500 mM NaCl, 20 mM imidazole, and 10% glycerol), and the bound protein was eluted using elution buffer (50 mM Tris-HCl, pH 8.0, 500 mM NaCl, 250 mM imidazole, and 10% glycerol). For purification of maltose-binding protein (MBP)-fused proteins, Amylase Resin (NEB, Ipswich, MA, USA) was used with MBP column buffer (50 mM Tris-HCl, pH 8.0, 200 mM NaCl). Elution was performed using MBP elution buffer containing 50 mM Tris–HCl (pH 8.0), 200 mM NaCl, and 10 mM maltose.

### 4.4. Size Exclusion Chromatography (SEC) and Size Prediction of Proteins

Proteins were separated using fast protein liquid chromatography (FPLC) with FPLC buffer (100 mM Tris-HCl, pH 8.0, 150 mM NaCl) on an ÄKTA purifier (Cytiva, Marlborough, MA, USA) equipped with a HiPrep™ 26/60 Sephacryl^®^ S-300 HR column (Cytiva, Marlborough, MA, USA). Protein elution was monitored by absorbance at 280 nm. The molecular sizes of the eluted proteins were estimated using a standard curve calibrated with the Gel Filtration Standard (Bio-Rad, Hercules, CA, USA).

### 4.5. Measurement of Absorption Spectrum

Eluted proteins were concentrated to an absorbance of 2.5 at 280 nm using an Amicon^®^ Ultra-4 Centrifugal Filter Unit (Merck KGaA, Darmstadt, Germany). Absorption spectra were collected using approximately 4.2 mg/mL protein in FPLC buffer (100 mM Tris–HCl, pH 8.0, 150 mM NaCl). A survey scan was performed at 5 nm increments across a wavelength range of 260–650 nm using an OPTIZEN™ POP spectrophotometer (Optizen, Daejeon, Republic of Korea). The values were collected two or three times.

### 4.6. Sequence Alignment and Structural Prediction of Proteins

The multiple sequence alignment was generated using Clustal Omega (https://www.ebi.ac.uk/jdispatcher/msa/clustalo, accessed on 5 February 2025) and visualized using JalView (Version 2.11.4.1) [49]. A three-dimensional model of the AtIBR5 sequence was generated using the molecular replacement method with the Phyre2 server, employing 1YUX from the Protein Data Bank as a template [50]. The predicted structures were analyzed and visualized using ChimeraX (Version 1.9) [51].

### 4.7. Inductively Coupled Plasma–Mass Spectrometry (ICP-MS)

Recombinant proteins were sequentially purified using affinity purification followed by SEC. The protein fractions predicted to contain AtIBR5 dimers were concentrated using an Amicon^®^ Ultra-4 Centrifugal Filter Unit (Merck KGaA, Darmstadt, Germany). Metal content was analyzed using a NexION 2000 (PerkinElmer, Waltham, MA, USA) after pretreatment of the protein samples. The values collected twice were averaged.

### 4.8. Immunoprecipitation and Immunoblot Experiment

The powdered plant material was mixed with 4 mL of extraction buffer (25 mM Tris-HCl, pH 7.5, 1 mM EDTA, 150 mM NaCl, 10% glycerol, 1× PMSF, and cOmplete™ Protease Inhibitor Cocktail [Merck KGaA, Darmstadt, Germany]) and incubated for 15 min. The sample was centrifuged at 3500× *g* for 15 min at 4 °C. The supernatant was filtered through Miracloth (Merck KGaA, Darmstadt, Germany) and re-centrifuged at 14,000× *g* for 20 min at 4 °C. Triton X-100 was added to the supernatant to a final concentration of 1%, followed by the addition of 30 μL of Anti-FLAG resin (Merck, Germany). Samples were incubated overnight at 4 °C. After washing five times with extraction buffer, the samples were boiled. Immunoblot experiments were performed as described by Yeon et al. [52]. The experiments were repeated three times.

### 4.9. Measurement of Hypocotyl Length, Primary Root Length, Leaf Serration, and Petal Size

Primary root length was measured using plants grown on 1/2 MS+V solid medium (2.2 g Murashige and Skoog with vitamins [Duchefa, Haarlem, The Netherlands], 0.05% 2-Morpholinoethanesulfonic acid [Duchefa, Haarlem, The Netherlands], 1% sucrose, and 1.2% Phyto Agar [Duchefa, Harlem, The Netherlands]) in the absence or presence of 100 nM IAA. To investigate auxin-induced root inhibition, 10-day-old plants grown on 1/2 MS+V solid medium, either without or with 100 nM IAA, were used. To assess ABA-induced root inhibition, 3-day-old seedlings germinated on 1/2 MS+V solid medium were transferred to fresh 1/2 MS+V solid medium, either without or with 10 μM ABA, and grown for an additional 4 days. To measure root length, plants were grown on plates placed in a vertical position. The degree of serration in atibr5 transgenic plants expressing AtIBR5 with or without the functional rubredoxin-like domain was determined by comparing the actual leaf area with the area of an imagined elliptical shape created by connecting the serrated edges (Figure 5b). Hypocotyl length, primary root length, leaf area, and petal size—both length and width—were measured using corresponding images and the ImageJ program (Version 1.54g). The measurements for each phenotype were conducted in triplicate, except for hypocotyl and root lengths, which were measured twice.

### 4.10. Observation of Cotyledon Vein Pattern

To determine whether the rubredoxin-like domain is also required for normal cotyledon vein development, relative frequencies of various vein patterns of ten-day-old seedlings were determined for WT, atibr5, atibr5+FL, atibr5+p, and atibr5+q plants. To this end, seedlings grown on 1/2 MS+V solid media were treated with 75% ethanol and 25% acetic acid overnight. The seedlings were then washed for one hour each in 70%, 80%, 90%, and 100% ethanol. After washing, they were treated with a 50% glycerol solution overnight. Vein patterns were classified into nine categories, as in Yanagisawa et al. [38], based on complexity and disconnection. Statistical analysis was performed on the percentages of each cotyledon vein pattern using a one-way ANOVA, followed by Tukey’s post hoc test. Three batches of experiments were conducted, using an average of 48 to 78 seedlings per genotype.

## 5. Conclusions

We report that AtIBR5 contains a metal-binding N-terminal rubredoxin-like domain, which is required for various, but not all, normal auxin and ABA responses in Arabidopsis. To better understand the role of the N-terminal rubredoxin-like domain in AtIBR5, a comprehensive investigation of the interaction partners of AtIBR5 and molecular outcomes of specific interactions in the presence of absence of the N-terminal rubredoxin-like domain are necessary.

## Figures and Tables

**Table 1:** Metal contents in recombinant proteins purified via Ni–NTA affinity chromatography.

Ion	SUMO-AtIBR5	SUMO-AtIBR5-p	SUMO-AtIBR5-q	GmDES1
Zn	3874 ± 452.14 (48.36)	271 ± 27.57 (5.07)	347 ± 67 (7.14)	554 ± 15.0 (10.96)
Ni	3603 ± 400.30 (44.98)	4553 ± 559.97 (85.10)	4122 ± 396.37 (84.40)	4113 ± 541.44 (80.63)
Cu	460 ± 79.2 (5.74)	471 ± 127.89 (8.72)	359 ± 90.51 (7.42)	377 ± 60.53 (7.37)
Mg	53 ± 26.03 (0.66)	38 ± 19.84 (0.73)	30 ± 0.35 (0.63)	33 ± 10.41 (0.65)
Fe	<10 (0.12)	<10 (0.19)	<10 (0.21)	<10 (0.2)
Cd	<10 (0.12)	<10 (0.19)	<10 (0.21)	<10 (0.2)

The values are presented in parts per billion (ppb) and represent the average of two independent samples. Numbers in parentheses indicate the percentage of each metal relative to the total metal content for each protein. For percentage calculations, a minimum value of 10 ppb was assigned for Fe (iron) and Cd (cadmium). The metal ion content detected in the purification buffer was as follows (Figure S5): 12.05% for Zn, 21.94% for Ni, 15.30% for Mg, and 50.71% for Fe.

**Table 2:** Metal contents in MBP-AtIBR5 and MBP-AtIBR5-q, purified via amylose affinity chromatography.

Ion	MBP-AtIBR5	MBP-AtIBR5-q
Zn	3034 (75.74)	439 (28.07)
Mg	357 (8.91)	494 (31.59)
Ni	279 (6.96)	298 (19.05)
Cu	181 (4.52)	174 (11.13)
Fe	148 (3.69)	151 (9.65)
Mn	7 (0.17)	8 (0.51)

Numbers in parentheses indicate the percentage of each metal relative to the total metal content for each protein.

## Data Availability

The data presented in this study are available on request from the corresponding author.

## Supplementary Materials

The following supporting information can be downloaded at https://www.mdpi.com/article/10.3390/ijms26199315/s1.

## Abbreviations

The following abbreviations are used in this manuscript:

Arabidopsis	*Arabidopsis thaliana*
*Arabidopsis thaliana* AtIBR5	*Arabidopsis thaliana* indole-3-butyric acid response 5
IAA	indole-3-acetic acid
DSP	dual-specificity phosphatase
IBA	indole-3-butyric acid
ABA	abscisic acid
WT	wild type
SEC	size exclusion chromatography
ICP-MS	inductively coupled plasma mass spectrometry
