# Supplementary material for: N-Terminal Metal-Binding Domain of Arabidopsis IBR5 Is Important for Its in Planta Functions
Source: Int J Mol Sci. 2025 Sep 24;26(19):9315. doi: 10.3390/ijms26199315 (PMC12525521; doi:10.3390/ijms26199315)
Supplement: Supplementary file 1 [file ijms-26-09315-s001.zip › SupplementaryFigureCaption.pdf]

Figure captions for supplementary Figures.

Figure S1. Recombinant SUMO-AtIBR5 protein purified using Ni-NTA affinity chromatography exhibits a brownish color. (A–C) Microcentrifuge tubes containing the elution buffer for affinity purification, SUMO-AtIBR5 in the elution buffer, and SUMO-AtIBR5-p in the elution buffer, respectively. SUMO-AtIBR5, SUMO-AtIBR5-p, and SUMO-AtIBR5-q are proteins with an N-terminal 6X His-SUMO tag and a glycine linker, translationally fused to wild-type AtIBR5, an N-terminally truncated AtIBR5, and a quadruple AtIBR5 mutant, respectively. In SUMO-AtIBR5-q, the quadruple AtIBR5 mutant, all four cysteine residues conserved in the rubredoxin-like domain—C10, C13, C25, and C28—were substituted with glycine. **Figure S2**. The four cysteine residues in AtIBR5 are positioned at suitable distances for metal coordination. (A) Sequence alignment of segments from AtIBR5, 3PZA, and 1YUX. The four conserved cysteine residues in each protein are highlighted in bold red. (B) A three-dimensional model of the AtIBR5 sequence (amino acids 8–28), generated based on the 1YUX structure. The iron atom detected in 1YUX is represented by a red sphere. The positions of the four conserved cysteine residues in AtIBR5—C10, C15, C25, and C28—are indicated in the model. **Figure S3.** SDS-PAGE analysis of purified proteins. (A) Recombinant proteins fused to an N-terminal 6X His tag were purified in two steps: first by Ni-NTA affinity chromatography, followed by size-exclusion chromatography (SEC). (B) Recombinant proteins fused to a maltose-binding domain (MBP) were purified in two steps: first by amylose affinity chromatography, followed by SEC. **Figure S4**. Percentages of metals bound to various proteins and present in the size-exclusion chromatography (SEC) buffer. The percentages were calculated based on the ppb values presented in Table 1, which were obtained from two experiments. SUMO-AtIBR5, SUMO-AtIBR5-p, and SUMO-AtIBR5-q are recombinant proteins carrying an N-terminal 6X His-SUMO tag and a glycine linker, translationally fused to wild-type (WT) AtIBR5, an N-terminally truncated AtIBR5, and a quadruple AtIBR5 mutant, respectively. In SUMO-AtIBR5-q, all four conserved cysteine residues in SUMO-AtIBR5 were substituted with glycine. GmDES1: *Glycine max* cysteine desulfhydrylase 1, carrying an N-terminal 6X His tag. Buffer: SEC buffer. **Figure S5**. FLAG-tagged proteins are well expressed in various *ibr5* transgenic lines. Western blot analysis of FLAG-tagged AtIBR5 proteins in WT and transgenic plants. M: Protein molecular marker. WT (Col-0): Wild-type Arabidopsis. *atibr5*: *atibr5* mutant (Salk_039359). *atibr5+FL*: *atibr5* mutant expressing full-length AtIBR5. *atibr5+p*: atibr5 mutant expressing only the DSP_plant_IBR5-like phosphatase domain of AtIBR5. *atibr5+q*: *atibr5* mutant expressing a mutant form of AtIBR5, in which the conserved four cysteine residues are substituted with glycines. The 25 kDa bands correspond to primary antibody chains conjugated with agarose resin. **Figure S6**. Representative cotyledon vein patterns for classification. In each image, the number before the dash (–) represents the number of closed areoles, while the number after the dash indicates the number of free-ending basal strands. Vein disconnections are classified into three categories: open distal areoles (ODA), basal free ends (BFE), and basal gaps (BGA). **Figure S7**. Amino acid sequence alignment of AtIBR5 and its orthologs from 38 plant species in the PANTHER database. Sequences are represented by five-letter codes, consisting of the first three letters of the genus name followed by the first two letters

of the species name. The following abbreviations were used: AMBTR for *Amborella trichopoda* (UniProt ID: W1P1V1), ARATH for *Arabidopsis thaliana* (UniProt ID: Q84JU4), BRADI for *Brachypodium distachyon* (UniProt ID: I1GYS6), BRANA for *Brassica napus* (UniProt ID: I1GYS6), BRARA for *Brassica rapa subsp. pekinensis* (UniProt ID: M4D9P1), CAPAN for *Capsicum annuum* (UniProt ID: A0A1U8E2D0), CHLRE for *Chlamydomonas reinhardtii* (UniProt ID: A8IWH5), CITSI for *Citrus sinensis* (UniProt ID: A0A067F8R7), CUCSA for *Cucumis sativus* (UniProt ID: A0A0A0KJP0), ERYGU for *Erythranthe guttata* (UniProt ID: A0A022RTT1), EUCGR for *Eucalyptus grandis* (UniProt ID: A0A058ZYM7), GLYMA for *Glycine max* (UniProt ID: I1M7K1), GOSHI for *Gossypium hirsutum* (UniProt ID: A0A1U8K1J4), HELAN for *Helianthus* (UniProt ID: A0A251TLX6), JUGRE for *Juglans regia* (UniProt ID: A0A2I4HX44), KLENI for *Klebsormidium nitens* (UniProt ID: A0A1Y1HPI8), LACSA for *Lactuca sativa* (UniProt ID: A0A9R1VMI2), MANES for *Manihot esculenta* (UniProt ID: A0A2C9UP75), MARPO for *Marchantia polymorpha annuus* (UniProt ID: A0A2R6X698), MEDTR for *Medicago truncatula* (UniProt ID: G7K7F8), MUSAC for *Musa acuminata subsp. malaccensis* (UniProt ID: A0A804KLK5), NELNU for *Nelumbo nucifera* (UniProt ID: A0A1U7ZVU5), NICTA for *Nicotiana tabacum* (UniProt ID: A0A1S3XV98), ORYSA for *Oryza sativa subsp. japonica* (UniProt ID: Q0DCN6), PHYPA for *Physcomitrium pate ns* (UniProt ID: A0A2K1K4D7), POPTR for *Populus trichocarpa* (UniProt ID: B9IAM0), PRUPE for *Prunus persica* (UniProt ID: M5WTJ1), RICCO for *Ricinus communis* (UniProt ID: B9SSI3), SETIT for *Setaria italica* (UniProt ID: K3XYS2), SOLLY for *Solanum lycopersicum* (UniProt ID: A0A3Q7J1T2), SOLTU for *Solanum tuberosum* (UniProt ID: M1B3A1), SORBI for *Sorghum bicolor* (UniProt ID: C5XZU5), SPIOL for *Spinacia oleracea* (UniProt ID: A0A9R0JYK7), THECA for *Theobroma cacao* (UniProt ID: A0A061DKZ4), TRIAE for *Triticum aesativum* (UniProt ID: A0A3B6SCF4), VITVI for *Vitis vinifera* (UniProt ID: D7TEG6), ZEAMA for *Zea mays* (UniProt ID: C0PIK7), and ZOSMA for *Zostera marina* (UniProt ID: A0A0K9PW53). The more conserved amino acids are highlighted in darker blue. The conserved motifs, including the catalytic cysteine and arginine within the DSP domain, are indicated by a red box, while the positions of four conserved cysteine residues present in all IBR5 sequences shown here are marked with red upside-down triangles. **Figure S8**. Amino acid sequence alignments of AtIBR5 and similar proteins in some algae and an organism outside green plant lineage. Sequences from various organisms in the NCBI protein database were indicated by 5 letter codes, three letters from the genus name followed by two letters from species name. ARATH: NP_178534.2 in *Arabidopsis thaliana*. TRESP: KAA6427192.1 in *Trebouxia sp. A1-2*, a Chlorophyta green alga (taxid: 3041). CHABR: GBG61469.1 from *Chara braunii*, a Charophyta green alga (taxid: 304574). RHOMA: KAJ8902311.1 in *Rhodosorus marinus*, a red alga (taxid: 2763). ECTSI: CBJ26669.1 in *Ectocarpus siliculosus*, a brown algae (taxid: 2870). PHRPL: KAH0629715.1 in *Phrynosoma platyrhinos*, desert horned lizard outside green plant lineage (taxid: 33090). The more conserved amino acids were shown with darker blue colors. The conserved motifs, including the catalytic cysteine and arginine within the DSP domain, are indicated by a red box, while the positions of four cysteine residues conserved across all IBR5 proteins in Figure S8 are marked with red upside-down triangles. The Arabidopsis sequence is marked with an asterisk.
